# Regulation of Eicosanoid Pathways by MicroRNAs

**DOI:** 10.3389/fphar.2019.00824

**Published:** 2019-07-19

**Authors:** Meike J. Saul, Anne C. Emmerich, Dieter Steinhilber, Beatrix Suess

**Affiliations:** ^1^Department of Biology, Technische Universität Darmstadt, Darmstadt, Germany; ^2^Institute of Pharmaceutical Chemistry, Goethe Universität Frankfurt, Frankfurt, Germany

**Keywords:** microRNA, new miRNA functions, eicosanoids, inflammation, prostaglandins

## Abstract

Over the last years, many microRNAs (miRNAs) have been identified that regulate the formation of bioactive lipid mediators such as prostanoids and leukotrienes. Many of these miRNAs are involved in complex regulatory circuits necessary for the fine-tuning of biological functions including inflammatory processes or cell growth. A better understanding of these networks will contribute to the development of novel therapeutic strategies for the treatment of inflammatory diseases and cancer. In this review, we provide an overview of the current knowledge of miRNA regulation in eicosanoid pathways with special focus on novel miRNA functions and regulatory circuits of leukotriene and prostaglandin biosynthesis.

## Introduction

MicroRNAs (miRNAs) are a family of small non-coding RNAs that regulate a wide range of biological processes including cancer development (Garzon et al., 2006; Croce, 2009; Ochs et al., 2014). In 1993, lin-4 was the first miRNA to be discovered in the nematode *Caenorhabditis elegans* (*C. elegans*) and it was found to regulate the gene lin-14 on the post-transcriptional level during *C. elegans* development (Wightman et al., 1993). Later, a second small miRNA involved in worm development, let-7, was identified (Reinhart et al., 2000). However, at the time of discovery, it was assumed that these RNAs were rare exceptions and only present in nematodes. In 2001, three independent publications reported the existence of several hundreds of these small non-coding RNAs not only in nematodes but also in murine and human cells (Lagos-Quintana et al., 2001; Lau et al., 2001; Lee and Ambros, 2001). Currently, more than 2,500 human miRNAs have been identified (http://www.mirbase.org), although the functions of many of them are still unknown. In this review, we will give a short overview of novel miRNA functions involved in inflammatory processes. Moreover, we summarize the recent findings on miRNAs regulating key enzymes of the eicosanoid signaling pathway.

## Biogenesis and Functions of MIRNAs

**Figure 1:**
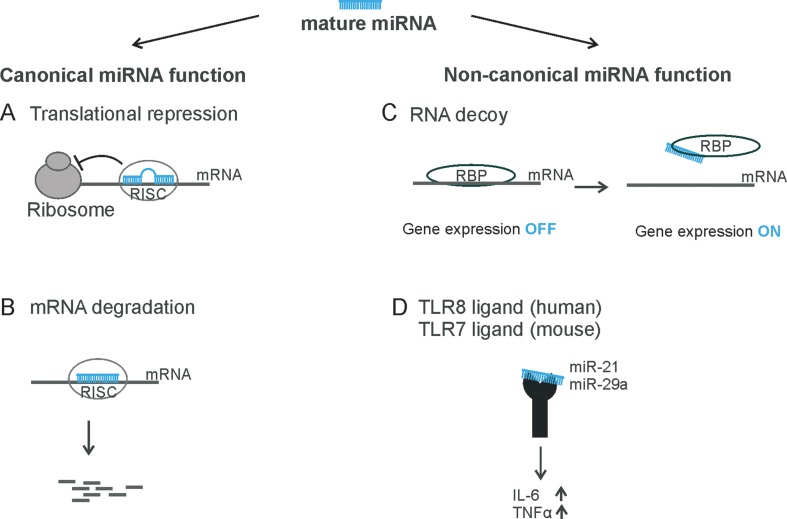
Overview of miRNA functions. The mature miRNA can be incorporated into the RNA-induced silencing complex (RISC) and binds to its target mRNA **(A)** to repress translation or **(B)** to induce mRNA degradation—for example, endonucleolytic cleavage. **(C)** miRNAs can act as RNA decoy to RNA-binding proteins, such as miR-328 to hnRNP E2 or miR-574-5p to CUGBP1. **(D)** miRNAs can be recognized by toll-like receptors 7/8, like miR-21 and miR-29a.

MiRNAs are transcribed from genes as long primary transcripts (pri-miRNAs) mostly by RNA polymerase II. In the nucleus, these pri-miRNAs are subsequently cleaved by the endonuclease Drosha. Drosha generates about 70 nucleotide (nt) long precursors (pre-miRNAs) that form imperfect stem-loop structures. Pre-miRNAs are transported out of the nucleus by exportin-5. In the cytoplasm, they are subsequently processed by a multiprotein complex including the RNAse III Dicer, Argonaute 2 (AGO2), and trans-activation-responsive RNA-binding protein (TRBP) to cleave the RNA into short 21–24 nt miRNA duplexes. Only one strand of the duplex is incorporated into a ribonucleoprotein complex, known as RNA-induced silencing complex (RISC). The passenger strand is mostly rapidly degraded. The miRNAs are then directed to their binding sites, which are usually located in the 3′ untranslated region (UTR) of their target messenger RNA (mRNA). Subsequently, they mediate endonucleotic cleavage, translational repression, or deadenylation of the mRNA transcript, followed by decapping and degradation of the target mRNA (Filipowicz et al., 2008) (**Figures 1A, B**). Of note, 40% of the currently known miRNAs are located within introns (mirtrons) and are processed by the spliceosome in an alternative way (Kim and Kim, 2007; De Rie et al., 2017).

Traditionally, it has been assumed that miRNAs are loaded into RISC and bind to their target mRNA through specific base pairing and that they reduce gene expression at the post-transcriptional level as their sole canonical function. However, there is recent evidence that miRNAs are also able to activate gene expression *via* a non-canonical mechanism (**Figures 1C, D**). miRNAs can bind to RNA-binding proteins (RBPs) and sequester them from their target mRNAs in a RISC-independent manner. This was shown for miR-328, which acts as an RNA decoy for the heterogeneous nuclear ribonucleoprotein E2 (hnRNP E2), a global post-transcriptional regulator (Eiring et al., 2010; Saul et al., 2016) (**Figure 1C**). As a consequence, hnRNP E2 target gene expression is activated.

Recent findings demonstrate that cells can secrete bioactive molecules (like proteins, lipids, or nucleic acids) from cell to cell *via* extracellular vesicles. Those vesicles can be divided into small extracellular vesicles (sEVs, also known as exosomes), microvesicles or apoptotic vesicles distinguished by their size (Thery et al., 2018). Although these extracellular particles were traditionally considered to be a “disposal system” for unnecessary membrane proteins (Johnstone et al., 1991), they have now captured the interest of researchers as part of the cell to cell communication (Wen et al., 2017).

In fact, sEVs (<200 nm) are highly concentrated with miRNAs (Goldie et al., 2014). Different cell types, including immune and cancer cells, are capable of secretion and uptake of extracellular miRNAs from sEVs. This suggests that sEVs could be part of the intercellular communication and carry out novel biological functions. Since the extracellular miRNA content does not necessarily reflect the cellular miRNA profile of the recipient cell (Zhang et al., 2015), the functional analysis of sEV-delivered miRNAs is an interesting subject. In a study focusing on non-canonical miRNA functions, which are receptor-mediated, it was demonstrated that two sEV-delivered miRNAs, miR-21 and miR-29a, are able to bind to the murine toll-like receptor (TLR) 7 and the human TLR8 and induce cytokine expression (Fabbri et al., 2012). Similar results were found for miRNA let-7b in the context of the nervous system. Extracellular let-7b activates murine TLR7 and induces neurodegeneration. This observation is of particular interest because let-7b has been detected in the cerebrospinal fluid of patients with Alzheimer´s disease (Lehmann et al., 2012). Thus, sorting of miRNAS into EV and the interaction of EV-delivered miRNAs with target cell components such as certain receptors seems to be an important way of cell–cell communication.

An interesting aspect for further investigations in this context is to explore how miRNAs are sorted into EVs. Initial studies demonstrated that miRNAs are specifically recognized by RBPs, such as hnRNP A2/B1 and Y-box protein 1 (YBX1). Those RBPs bind sequence-specifically to miRNAs and load them selectively into sEVs (Villarroya-Beltri et al., 2013; Shurtleff et al., 2016). These data suggest that the loading of miRNAs into EV is a specific process that might control biological processes such as immune functions.

## miRNAs and Eicosanoids

**Figure 2:**
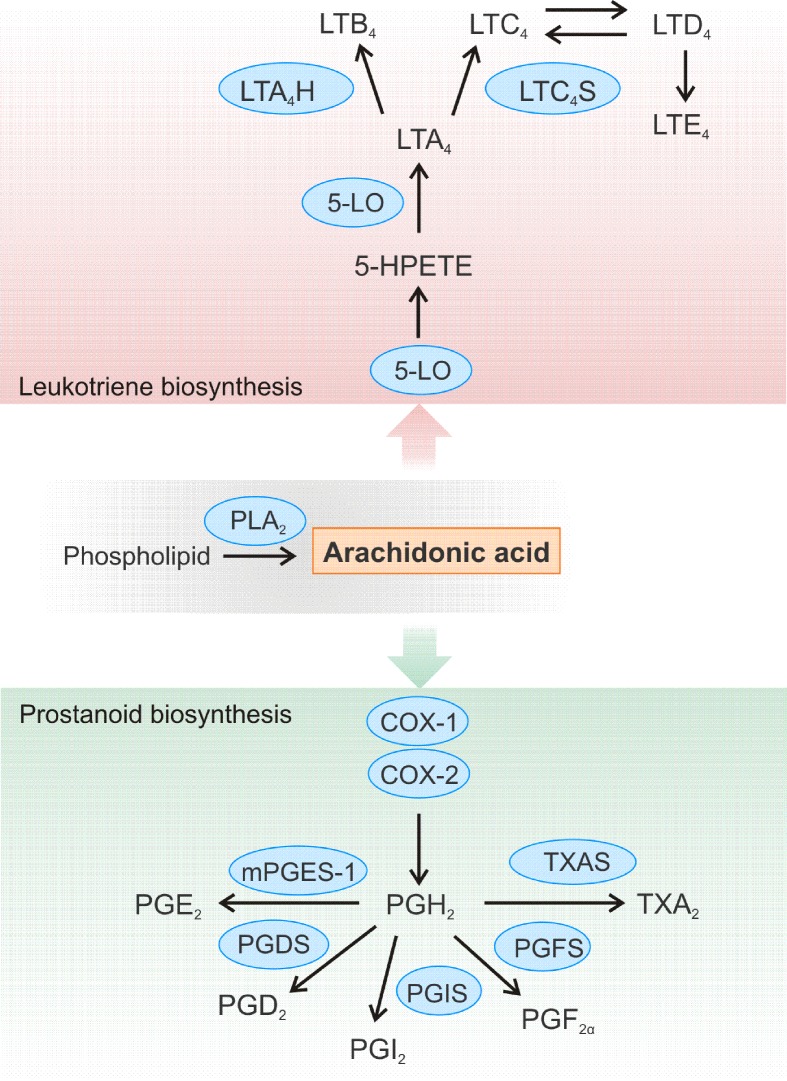
Schematic overview of the leukotriene and prostanoid biosynthesis pathway. Arachidonic acid (AA) is released from cellular membranes by cytosolic phospholipase A_2_ (PLA_2_). The free AA can further be converted to different leukotrienes (LT). 5-LO is crucial for the conversion of AA to 5(S)-hydroperoxyeicosatetraenoic acid (5-HPETE) and LTA_4_. LTA_4_ is further converted to LTB_4_ by LTA_4_ hydrolase (LTA_4_H) or to the cysteinyl-containing LTC_4_ by LTC_4_ synthase (LTC_4_S), which can be metabolized to LTD_4_ and LTE_4_. Biosynthesis of prostanoids begins with the enzyme cyclooxygenase (COX)-1 or COX-2 converting AA to prostaglandin (PG) H_2_, which is further converted to a variety of other prostanoids. Thromboxane A_2_ (TXA_2_) is generated by TXA synthase (TXAS), while the synthases PGxS produce the certain PGs: PGF_2α_, PGI_2_, and PGD_2_. The microsomal PGE synthase-1 (mPGES-1) catalyzes formation of PGE_2_.

Eicosanoids such as prostaglandins and leukotrienes are biologically active lipid mediators that are products of a local cell type-specific arachidonic acid (AA) metabolism (**Figure 2**). Such lipid mediators play a critical role in different pathological processes like inflammation and cancer (Zeldin, 2001; Wang and Dubois, 2010; Radmark et al., 2015). The synthesis of eicosanoids begins with the release of AA from the cell membrane by phospholipase A_2_ which is followed by the metabolism of the AA through cyclooxygenases (COXs), lipoxygenases (LOXs), and cytochrome P450 enzymes (Wang and Dubois, 2010). Due to the key role of these enzymes in the formation of bioactive lipid mediators, it is not surprising that 5-LO and COX enzymes are prominent miRNA targets (for previous reviews, see also Ochs et al., 2011; Ochs et al., 2014).

## miRNA Regulation in Prostanoid Biosynthesis

The group of prostanoids consists of prostaglandins, thromboxane A_2_, as well as prostacyclin. Their common denominator is that they can all be formed from AA, which is converted by COX enzymes in a two-step process to PGH_2_ (**Figure 2**). This intermediate is then converted to the different prostaglandins by the respective synthases. Prostanoids belong to the most important inflammatory signaling molecules. These lipid mediators exert their multiple biological effects in an autocrine and paracrine manner by binding to their specific cell surface G protein-coupled receptors. For example, prostaglandin E_2_ (PGE_2_) is a bioactive lipid that can elicit a wide range of biological effects associated with inflammation and cancer (Jakobsson et al., 1999; Pettersson et al., 2005; Nakanishi and Rosenberg, 2013). It contributes to the development of inflammation and plays a predominant role in promoting cancer progression by induction of cellular proliferation and tumor angiogenesis, inhibition of apoptosis, and suppression of immune responses (Wang and Dubois, 2006; Larsson and Jakobsson, 2015). PGE_2_ is formed by the conversion of AA to PGH_2_ by the cyclooxygenases COX-1 or COX-2, followed by processing by PGE synthases, of which microsomal prostaglandin E synthase 1 (mPGES-1) is the key enzyme. The formed PGE_2_ is then rapidly secreted to act on their specific receptors on recipient cells. These receptors are not only present on immune cells, but also on a variety of cells of the cardiovascular system like cardiomyocytes, smooth muscle cells, or vascular endothelial cells (Suzuki et al., 2011). Thus, PGE_2_ can be involved in the development of different cardiovascular diseases. It was shown that deletion of mPGES-1 impairs the left ventricular contractile function after acute myocardial infarction and leads to overall remodeling of the left ventricle (Degousee et al., 2008).

### Non-Canonical miRNA Regulation of Prostaglandin E_2_ Biosynthesis

A variety of cancer types exhibit increased levels of mPGES-1 and its enzymatic product PGE_2_—for example, colon (Yoshimatsu et al., 2001b; Wang and Dubois, 2010), prostate (Jain et al., 2008; Hanaka et al., 2009), lung (Yoshimatsu et al., 2001a), and breast cancer (Olesch et al., 2015). Interestingly, there were marked differences in the extent of upregulation of mPGES-1 and PGE_2_ in individual lung tumors (Yoshimatsu et al., 2001a; Wu et al., 2010). This effect can be attributed to individual expression variations of COX-2 and mPGES-1 (Wang et al., 2006; Hughes et al., 2008). However, the observed variability cannot be explained solely by different transcriptional regulation mechanisms [such as nuclear factor κB (NFκB)] (Yamamoto et al., 1995; Kang et al., 2007), suggesting that additional—namely, post-transcriptional mechanisms—might be involved—for instance, miRNA regulation.

Recently, we demonstrated that miR-574-5p activates mPGES-1-derived PGE_2_ synthesis in human non-small cell lung cancer (NSCLC). Moreover, this miRNA promotes tumor growth *in vivo*, which is completely blocked by the administration of the mPGES-1 inhibitor CIII (Leclerc et al., 2013). Mechanistically, it could be shown that miR-574-5p acts as RNA decoy to CUG-RNA-binding protein 1 (CUGBP1) (**Figures 1C** **,** **3A**) and that it antagonizes the CUGBP1 function as a post-transcriptional regulator (Mukhopadhyay et al., 2003; Subbaramaiah et al., 2003; Gao et al., 2015). In human, NSCLC as well as under inflammatory conditions, miR-574-5p is strongly upregulated and induces mPGES-1 expression by preventing CUGBP1 binding to the mPGES-1-3´UTR. This leads to an enhanced alternative splicing and the generation of a 3´UTR splice variant. Importantly, the nuclear localization of miR-574-5p and CUGBP1 is in line with its regulatory function on mPGES-1 mRNA splicing (Saul et al., 2019). The newly discovered association between miR-574-5p overexpression and PGE_2_-mediated growth of lung cancer cells *in vivo* suggests that pharmacological inhibition of PGE_2_ formation with mPGES-1 or COX inhibitors might be of considerable therapeutic value for NSCLC patients with high miR-574-5p levels (Saul et al., 2019). In this respect, mPGES-1 inhibition might be of particular interest since inhibition of mPGES-1, instead of COX-2, may be associated with fewer side effects as other prostanoids would not be affected.

**Figure 3:**
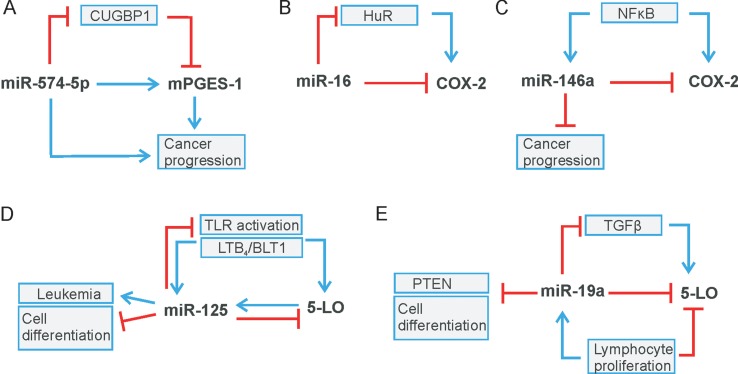
Regulatory circuits of leukotriene and prostaglandin biosynthesis. **(A)** miR-574-5p/CUGBP1 regulates mPGES-1 level, **(B)** miR-16/HuR and **(C)** miR-146a modulate COX-2 expression. **(D)** miR-125 and **(E)** miR-19a regulate 5-LO level. Stimulation is indicated by blue arrows, whereas red lines indicate inhibition.

### Canonical miRNA Regulation of Prostaglandin Biosynthesis

There are two known isoenzymes responsible for the generation of prostanoids: cyclooxygenase-1 (COX-1) and cyclooxygenase-2 (COX-2), both of which catalyze the same enzymatic reaction. COX-1 represents a housekeeping enzyme responsible for maintaining basal prostanoid levels, which are important for tissue homeostasis. In contrast, COX-2 is barely detectable in most normal tissues, but is strongly induced in response to inflammatory cytokines, hypoxia, and other stressors (Samuelsson et al., 2007; Wang and Dubois, 2010).

COX-2 expression is regulated at different levels (Dixon et al., 2000; Harper and Tyson-Capper, 2008). Various transcription factors such as NFκB, activator protein 1 (AP1), or the cAMP-responsive element-binding protein (CREB) are involved in its transcriptional regulation (Yamamoto et al., 1995; Kang et al., 2007).

At the post-transcriptional level, COX-2 expression is regulated by mRNA stability and translation efficiency mostly including *trans*- and *cis-acting* factors (Dixon et al., 2000; Young and Dixon, 2010). For example, RNA-binding proteins can interact with AU-rich elements (AREs) within the COX-2 3´UTR like CUG triplet repeat-RNA-binding protein 2 (CUGBP2) (Mukhopadhyay et al., 2003) or the mRNA-stability factor HuR (Erkinheimo et al., 2003; Subbaramaiah et al., 2003). Tristetraprolin (TTP), another RNA-binding protein that promotes mRNA instability, has been shown to bind to the 3´UTR of COX-2 and thus contributes to the post-transcriptional regulation of COX-2 (Sawaoka et al., 2003). In addition, the use of an alternative polyadenylation signal may regulate COX-2 mRNA stability and translation (Hall-Pogar et al., 2005; Hall-Pogar et al., 2007).

In recent years, miRNAs have been identified as additional players in the post-transcriptional control of COX-2 expression (see **Table 1**). Interestingly, miR-16 is complementary to sequences in the AU-rich regions of the COX-2 3’UTR, which allows direct binding of miR-16, and in turn, alters COX-2 mRNA stability (Jing et al., 2005) (**Figure 3**). In association with TTP, miR-16 can promote mRNA decay. However, TTP does not bind directly to miR-16 but interacts through the association with RISC components to form a complex with miR-16 and promote mRNA degradation (Jing et al., 2005). Furthermore, miR-16 silences COX-2 expression in hepatoma cells *via* two mechanisms: by binding directly to the COX-2 3′-UTR and by decreasing the levels of the RNA-binding protein HuR (Agra Andrieu et al., 2012) (**Figure 3B**). MiR-16 also competes with the RNA-binding protein heterogeneous nuclear ribonuclear protein K (hnRNP K) for binding to COX-2 3´UTR and thus modulates COX-2 expression on the post-transcriptional level (Shanmugam et al., 2008).

**Table 1:** Summary—miRNAs influencing the prostaglandin pathway by binding to mRNAs of key enzymes or interfering with RNA-binding protein CUGBP1 (see mPGES-1).

miRNA	Target	Tissue/disease	Reference
Hsa-miR-144	COX-2	Premature labor	(Li et al., 2016)
Hsa-miR-144-5p	COX-2	Periodontitis	(Li et al., 2019)
Hsa-miR-144	COX-2	Esophageal squamous cell cancer	(Shao et al., 2016)
Hsa-miR-26a			
Hsa-miR-26b	COX-2	Nasopharyngeal epithelial cancer	(Ji et al., 2010)
Hsa-miR-26b	COX-2	Oral lichen planus	(Danielsson et al., 2012)
Hsa-miR-26b	COX-2	Breast cancer	(Li et al., 2013)
Hsa-miR-216a-3p	COX-2	Colorectal cancer	(Wang et al., 2018)
Hsa-miR-30a	COX-2	Gastric cancer	(Liu et al., 2017)
Hsa-miR-146a	mPGES-2	Bone marrow-derived mesenchymal stem cells	(Matysiak et al., 2013)
Hsa-miR-146a	COX-2	COPD	(Sato et al., 2010)
Hsa-miR-146a	COX-2	Astrocytes	(Iyer et al., 2012)
Hsa-miR-146a	COX-2	Colon cancer	(Zhang et al., 2019)
Hsa-miR-146a	COX-2	Lung cancer	(Cornett and Lutz, 2014)
Hsa-miR-146a	COX-2	COPD	(Wang et al., 2015)
Mmu-miR-199a	COX-2	Mouse uterus, endometrial cancer	(Chakrabarty et al., 2007; Daikoku et al., 2008)
Mmu-miR-101a			
Hsa-miR-101	COX-2	Colon cancer	(Strillacci et al., 2009)
Hsa-miR-101	COX-2	Endometrial serous carcinoma	(Hiroki et al., 2010)
Hsa-miR-101a	COX-2	Mammary gland	(Tanaka et al., 2009)
Hsa-miR-101	COX-2	Gastric cancer	(He et al., 2012)
Hsa-miR-101	COX-2	Prostate cancer	(Hao et al., 2011)
Hsa-miR-101-3p	COX-2	Esophageal squamous cell cancer	(Gong et al., 2016)
Hsa-miR-16	COX-2	Cervical cancer	(Jing et al., 2005)
Hsa-miR-16	COX-2	Hepatocellular carcinoma	(Agra Andrieu et al., 2012)
Hsa-miR-16	COX-2	Monocytes (THP-1 cells)	(Shanmugam et al., 2008)
Hsa-miR-137	COX-2	Glioma	(Chen et al., 2012)
Hsa-miR-143	COX-2	Amnion mesenchymal cells	(Kim et al., 2011)
Hsa-miR-542-3p	COX-2	Colon cancer	(Moore et al., 2012)
Hsa-miR-574-5p	mPGES-1	Lung cancer (NSCLC)	(Saul et al., 2019)
Hsa-miR-21	15-PGDH	Cholangiocarcinoma	(Lu et al., 2014)
Hsa-miR-21	15-PGDH	Tongue squamous cell cancer	(He et al., 2016)
Hsa-miR-21	15-PGDH	Gastric cancer	(Li et al., 2019)
Hsa-miR-21	15-PGDH	Colon cancer	(Monteleone et al., 2019)
Hsa-miR-26a/b	15-PGDH	Cholangiocarcinoma	(Yao et al., 2015)
Hsa-miR-620	15-PGDH	Prostate adenocarcinoma cell line (DU145); breast cancer cell line (MDA-MB-231)	(Huang et al., 2015)
Hsa-miR-218	15-PGDH	Synovial mesenchymal stem cells (SMSCs)	(Cong et al., 2017)
Hsa-miR146b-3p	15-PGDH	Cervical cancer	(Yao et al., 2018)

In addition, a considerable number of reports focus on miR-144, which directly targets COX-2 mRNA to downregulate its protein level. For example, the balance between miR-144, COX-2, and c-fos regulates PGE_2_ synthesis in the amnion of pregnant humans and mice. The amnion is the major source of PGE_2_ and plays a central role in the process of premature labor. The transcription factor c-fos induces expression of COX-2 and miR-144. The latter in turn generates a negative feedback loop by directly and indirectly inhibiting both COX-2 and c-fos. This inhibition has a negative effect on PGE_2_ generation and thus prevents premature contractions (Li et al., 2016). A negative correlation of miR-144-5p and COX-2 was also observed in human inflamed gingival tissue from periodontitis patients (Li et al., 2019). Moreover, a significant downregulation of miR-144 and miR-26a compared to healthy surrounding tissue was found in the tumor tissue of patients with esophageal squamous cell carcinoma (ESCC). The two miRNAs were confirmed to bind to COX-2 mRNA, thus downregulating the protein level of COX-2. It has been shown that, due to downregulation of miR-144 and miR-26a in tumor tissue, COX-2 activity significantly increased and subsequently promoted cell proliferation and metastasis (Shao et al., 2016). COX-2 is also a canonical target of miR-101-3p that is downregulated in ESCC cells stimulated with cigarette smoking extract (CSE). It has been shown that the promoting effect of CSE on ESCC is due to COX-2 upregulation. It was further demonstrated that the mechanism by which CSE regulates COX-2 expression is mediated by miR-101-3p to promote cell proliferation (Gong et al., 2016). Another miRNA that regulates COX-2 *via* its canonical function is miR-146a, a miRNA that is regulated like COX-2 by NFκB signaling (Poligone and Baldwin, 2001; Taganov et al., 2006). In human lung cancer, miR-146a directly regulates COX-2 mRNA and thus the protein level of COX-2 in lung cancer cells (Cornett and Lutz, 2014) (**Figure 3C**). In human fibroblasts of smokers with chronic obstructive pulmonary disease, it was found that single nucleotide polymorphisms in the miR-146a precursor caused several patients to have reduced miRNA level, which significantly improved baseline lung function (Wang et al., 2015). Very recently, it was demonstrated that the polymorphisms of miR-146a (rs2910164) and plasmacytoma variant translocation 1 (PVT1; rs13281615) affect the prognosis of colon cancer by regulating COX-2 expression and cell apoptosis. The presence of PVT1 decreased the expression level of miR-146a, which in turn increased the COX-2 level (Zhang et al., 2019). Another miRNA that suppresses inflammation-related tumors is miR-30a. MiR-30a is crucial for regulation of growth and migration of *Heliobacter pylori*–infected gastric cancer *via* targeting COX-2 and B cell CLL/lymphoma 9 (BCL 9) (Liu et al., 2017). In the same way, colorectal cancer is influenced by miR-216a-3p, which directly targets COX-2 *via* its canonical function (Wang et al., 2018).

In addition to the miRNA regulation of enzymes involved in PGE_2_ formation like COX-2 and mPGES-1, it is important to explore how the PGE_2_-metabolizing enzyme is regulated by miRs. The key enzyme that converts PGE_2_ to its biologically inactive metabolite is the NAD^+^-dependent 15-hydroxyprostaglandin dehydrogenase (15-PGDH/HPGD) (Ensor and Tai, 1995). In cholangiocarcinoma cells, 15-PGDH was identified as a direct target of miR-21. In addition, COX-2 overexpression and PGE_2_ treatment increase the level of miR-21 associated with enhanced miR-21 promoter activity (Lu et al., 2014). The finding that miR-21 directly regulates 15-PDGH was further confirmed by recent publications on tongue squamous cell carcinoma (He et al., 2016), gastric cancer (Li et al., 2019) and colon cancer (Monteleone et al., 2019). Interestingly, epithelial growth factor (EGF) signaling in colorectal cancer cells reduces the level of 15-PGDH and simultaneously increases the miR-21 level (Monteleone et al., 2019).

In addition to this, other miRNAs are known to modulate 15-PGDH expression. Omega-3 polyunsaturated fatty acids (ω-3 PUFA) upregulate 15-PGDH expression by inhibiting miR-26a and miR-26b. This directly contributes to ω-3 PUFA-induced inhibition of human cholangiocarcinoma cell growth, providing a preclinical justification for the evaluation of ω-3 PUFA in the treatment of cholangiocarcinoma (Yao et al., 2015). MiR-620 directly targets 15-PGDH, which results in an increase in PGE_2_ promoting radioresistance in cancer cells like the human prostate adenocarcinoma cell line DU145 and the breast cancer cell line MDA-MB-231 (Huang et al., 2015). Furthermore, miR-218 directly regulates the expression level of 15-PGDH during differentiation of synovial mesenchymal stem cells (SMSCs) into cartilage and subsequently inhibits osteogenesis during chondrogenesis (Cong et al., 2017). Finally, 15-PDGH was identified as the canonical target of miR-146b-3p that promotes proliferation, migration, and anchorage-independent growth of cervical cancer cells (Yao et al., 2018).

### Influence of the Prostaglandin Pathway on miRNA Expression

In addition to the regulation of the enzymes of the prostaglandin pathway by miRNAs, the influence of prostaglandin signaling on miRNA expression has also been investigated. It was found that COX-2 signaling increases the oncogenic miR-526b and miR-655 levels in human breast cancer by activating the EP4 receptor. Thus, COX-2 signaling strongly influences the phenotype of the tumor by promoting cellular migration, invasion, or proliferation (Majumder et al., 2015; Majumder et al., 2018).

In addition, it was found that cancer-associated fibroblasts (CAFs) develop a senescence-associated secretory phenotype (SASP) that contributes to cancer progression. Interestingly, senescent CAFs have increased levels of PGE_2_ and COX-2. Moreover, miR-335 is upregulated in the senescent normal fibroblasts as well as CAFs. This modulates the secretion of SASP factors and induces the mobility of cancer cells in co-cultures, at least partially by suppressing the expression of the phosphatase and tensin homologue (PTEN). With the application of the COX-2 inhibitor celecoxib, expression of miR-335 was strongly reduced, suggesting a new miR-335/COX-2/PTEN axis that modulates the inflammasome in senescent fibroblasts (Kabir et al., 2016).

In line with these results, it was demonstrated that the mPGES-1/PGE_2_ pathway affects the expression level of miR-15a and miR-186 in prostate cancer. High PGE_2_ levels reduced Dicer expression and consequently miRNA biogenesis in prostate cancer cells. It is noteworthy that PGE_2_-mediated downregulation of miR-15a and miR-186 is directly associated to vascular endothelial growth factor (VEGF) expression and angiogenesis. This suggests that these miRNAs may be potential candidates for mitigating the aggressive properties of prostate cancer. This alternative approach could overcome the chemo-resistance, which is common for drugs targeting VEGF and/or VEGF receptors (Terzuoli et al., 2016).

## miRNA Regulation in Leukotriene Biosynthesis

The 5-lipoxygenase (5-LO) is the key enzyme of the leukotriene pathway. It interacts with the 5-LO-activating protein (FLAP) and catalyzes the conversion of AA into 5(*S*)-hydroperoxyeicosatetraenoic acid (5-HPETE) and leukotriene (LT) A_4_. The LTA_4_ is then subsequently converted either into biologically active LTB_4_ by LTA_4_ hydrolase or into LTC_4_ by LTC_4_ synthase and the LTC_4_ synthase isoenzyme MGST2. Over the last decades, different studies have shown that 5-LO-derived AA metabolites play an important role in inflammatory reactions like inflammatory disorders and allergic diseases such as asthma, allergic rhinitis, cardiovascular diseases, as well as in different types of cancer (Steinhilber and Hofmann, 2014).

### Canonical miRNA Regulation on Leukotriene Pathway

In contrast to COX-2, the knowledge about post-transcriptional regulation of 5-LO is rather limited. It is known that the combination of alternative splicing and RNA decay modulates 5-LO gene expression (Ochs et al., 2012). Furthermore, several publications demonstrated that 5-LO is a canonical target for miR-19a-3p, miR-125-5p (Busch et al., 2015), miR-216-3p (Wang et al., 2018), and miR-674-5p (Su et al., 2016) (see **Table 2**). Specifically, miR-19a-3p and miR-125-5p regulate 5-LO expression in the human myeloid cell line MonoMac 6. In contrast, only miR-19a-3p modulates the 5-LO protein level in human T-lymphocytes stimulated with phytohemagglutinin (PHA). Overall, it reveals that miR-19a-3p and miR-125b-5p target 5-LO in a cell type and stimulus-specific manner (Busch et al., 2015). Interestingly, miR-125 and miR-19a seem to be parts of regulatory circuit-controlling immune reactions and cell proliferation (**Figures 3D, E**). 5-LO expression is induced by TLR/NFκB activation (Lee et al., 2015) or during cell stimulation by transforming growth factor (TGF)-β (Steinhilber et al., 1993). The same signals were also reported to induce miR-125 (Curtale et al., 2018; Hildebrand et al., 2018) as well as miR-19a, which in turn downregulates components of NFκB or TGF-β signaling, respectively, and also 5-LO (**Figures 3D, E**) (Dews et al., 2010; Busch et al., 2015). Thus, upregulation of miR-19a and downregulation of 5-LO expression are associated with cell proliferation in T-lymphocytes (Busch et al., 2015). Furthermore, it is well known that upregulation of miR-19a inhibits cell differentiation and promotes cell growth and cancer development—for instance, by suppression of PTEN (Lewis et al., 2003). MiR-674-5p was identified as a direct regulator of 5-LO mRNA in mice. It is further discussed as a negative regulator in 5-LO-mediated autoimmune diseases of the liver, thus representing a promising approach to future therapeutic measures (Su et al., 2016). Recently, it was shown that miR-216a-3p regulates not only COX-2 but also 5-LO expression in colon cancer, thus affecting colon cancer cell proliferation. These data indicate that miR-216-3p might represent a novel target for colorectal cancer treatment (Wang et al., 2018). Interestingly, not only 5-LO but also FLAP is targeted by miRs, like miR-146a in human lung cancer (Iacona et al., 2018). Moreover, miR-146a is also known to regulate COX-2 expression in lung cancer (Cornett and Lutz, 2014), which indicates a role of miR-146a as an endogenous dual inhibitor of AA metabolism in lung cancer by regulating prostaglandins and LTs, similar to miR-216a-3p (Iacona et al., 2018).

**Table 2:** Summary—miRNAs influencing the leukotriene pathway by binding to mRNAs of key enzymes.

miRNA	Target	Tissue/disease	Reference
Hsa-miR-19a-3p	5-LO	Monocytes (MM6 cells), T-lymphocytes	(Busch et al., 2015)
Hsa-miR-125-5p			
Hsa-miR-674-5p	5-LO	Acute mouse liver injury	(Su et al., 2016)
Hsa-miR-216a-3p	5-LO	Colorectal cancer	(Wang et al., 2018)
Hsa-miR-146a	FLAP	Lung cancer	(Iacona et al., 2018)
Hsa-miR-135a	FLAP	Pulmonary microvascular endothelial cells	(Gonsalves and Kalra, 2010)
Hsa-miR-199a			

### Influence of the Leukotriene Pathway on miRNA Expression

It is well known that 5-LO interacts with the C-terminal domain of human Dicer. The interaction between 5-LO and Dicer leads to an enhanced 5-LO enzymatic activity as well as Dicer activity *in vitro*. These results suggest that the processing of specific miRNAs by Dicer might be regulated by the 5‐LO/Dicer interaction (Dincbas-Renqvist et al., 2009). In addition to the direct 5-LO/Dicer interaction, 5-LO products can also modulate miRNA expression. Thus, LTB_4_ induces the expression of inflammatory miRNAs including miR-155, miR-146a and miR125b in macrophages *via* the LT B4 receptor-1 (BLT1) and Gα1 signaling (Wang et al., 2014).

## Conclusions

The number of miRNAs that were discovered in recent years to be involved in the regulation of the expression of key enzymes in prostanoid and LT biosynthesis is steadily increasing. **Tables 1**, **2** give a comprehensive overview of all currently known miRNAs involved in inflammatory processes. There is growing evidence that many of these miRNAs are involved in complex regulatory cascades and networks with multiple layers of integrated signals and feedback loops required for fine-tuning of biological functions such as inflammatory responses or cell growth. Besides the direct, canonical regulation of mRNA stability and translation of enzymes of the AA cascade by miRNAs, it was found that miRNAs that interfere with HUR or TTP function are involved in the regulation of COX-2 expression. Furthermore, a novel non-canonical mechanism was found for miR-574-5p, which acts as a decoy for CUGBP1 and strongly stimulates PGE_2_ formation. It becomes clear that miRNAs can also be packed into EVs so that they are involved in cell–cell communication. This suggests that miRNAs not only regulate cellular functions in an autocrine/intracrine manner but also can affect processes such as the formation of lipid mediators in a paracrine fashion. Of note, 5-LO was identified as a binding protein for Dicer, which suggests that 5-LO can modulate the Dicer function and interferes with miRNA generation. Thus, it becomes increasingly clear that there are multiple links between the miRNA network and lipid signaling. The progress in the understanding of these interactions will help to develop new therapeutic strategies for the treatment of inflammatory diseases and cancer.

## Author Contributions

MS wrote the manuscript. AE, DS, and BS contributed to writing and editing the manuscript.

## Funding

This project was supported by the Else Kröner-Fresenius Stiftung (Else Kröner-Fresenius-Graduiertenkolleg), the Deutsche Forschungsgemeinschaft (SFB902 and ECCPS), Athene Young Investigator program (Technische Universität Darmstadt; grant no: n/a), and BMBF (KMU-innovativ-22: miRTumorProst; 031B0768B).
